# Supplementary material for: Nuclear decoupling is part of a rapid protein-level cellular response to high-intensity mechanical loading
Source: Nat Commun. 2019 Sep 12;10:4149. doi: 10.1038/s41467-019-11923-1 (PMC6742657; doi:10.1038/s41467-019-11923-1)
Supplement: Supplementary file 1 — Supplementary Information [file 41467_2019_11923_MOESM1_ESM.pdf]

Supplementary Information

Nuclear decoupling is part of a rapid protein-level cellular response to high-intensity mechanical loading

Gilbert et al.

Supplementary Figures

Supplementary Fig. 1.

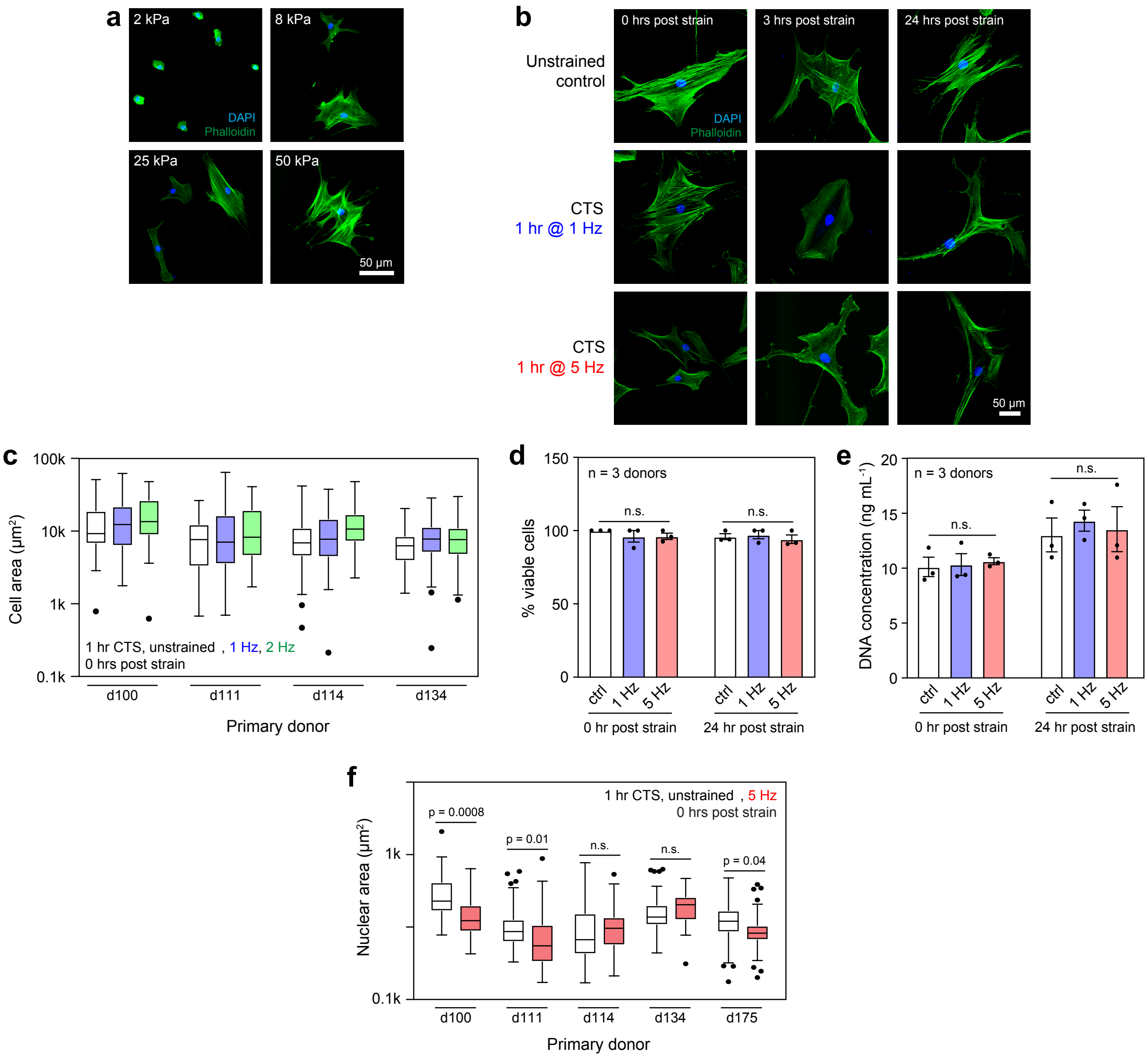

Supplementary Figure 1. Morphology and viability of primary human mesenchymal stem cells (hMSCs) subjected to cyclic tensile strain (CTS). (**a**) Representative images of hMSCs cultured for three days on collagen-I coated polyacrylamide hydrogels with stiffnesses between 2 and 50 kPa, with phalloidin and DAPI staining. (**b**) Representative images of hMSCs cultured for two days on collagen-I coated silicone elastomer BioFlex plates, followed by CTS as indicated, with phalloidin and DAPI staining. (**c**) Donor-by-donor analysis of cell spread areas of hMSCs subjected to 1 hour of CTS at 1 or 2 Hz (0 – 4% strain), compared to control cells. (**d**) hMSCs subjected to CTS (0 – 4% strain at 1 Hz, or 2.6 – 6.2% strain at 5 Hz, for 1 hour; $n = 3$ donors) showed no significant changes to viability, as determined by live/dead assay. Minimum numbers of cells analysed per condition for figure part (d): donor d105 used ≥16 cells per condition; d101, ≥11; d125, ≥12. (**e**) Quantification of DNA in hMSCs treated with CTS showed no significant changes to rates of proliferation. In plots (d) and (e), bars show mean and s.e.m.; dots represent data from individual donors; a lack of significant variation was determined from ANOVA testing. (**f**) Donor-by-donor analysis of nuclear areas of hMSCs following 1 hour of CTS at 5 Hz (2.6 – 6.2% strain), compared to control cells, p-values were determined from unpaired two-tailed t-tests. There was a significant decrease in nuclear area following strain in 3 out of 5 donors (d100, $p = 0.0008$; d111, $p = 0.01$; d175, $p = 0.04$). In (c) and (f), box-whisker plot centre lines show medians, bounds of box show quartiles, whiskers show data spread and outliers determined by the Tukey method. ≥14 cells analysed in donor d100, per condition; ≥31 cells analysed for d111, per condition; ≥40 cells analysed for d114, per condition; ≥42 cells analysed for d134, per condition; ≥33 cells analysed for d175, per condition.

Supplementary Fig. 2.

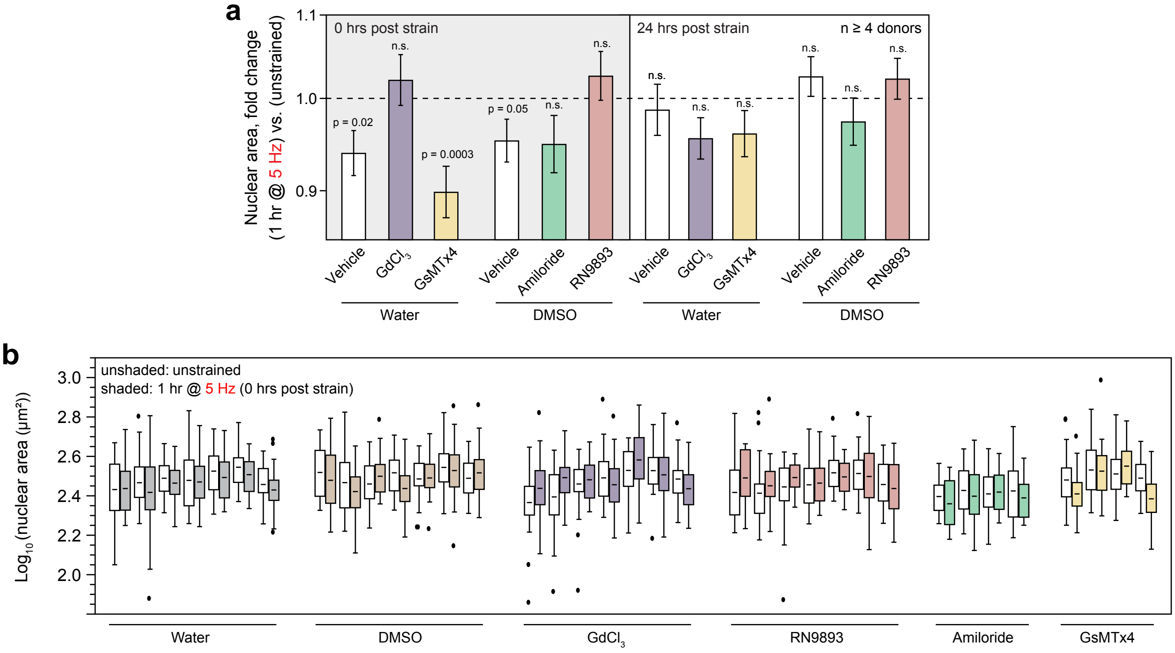

Supplementary Fig. 2. Role of ion channels in modulating responses to CTS in nuclear area. (**a**) Changes to nuclear areas in primary hMSCs immediately following CTS (1 hour, 2.6 – 6.2% strain at 5 Hz), normalized to unstrained controls. Ion channel activity was inhibited using: 10 μM $GdCl_3$, (n = 6 donors), a broad spectrum inhibitor of stretch-activated ion channels[1]; 3 μM GsMTx (n = 4 donors), a specific inhibitor of piezo channels[2]; 100 μM amiloride (n = 4 donors), an inhibitor of acid-sensing ion channels (ASICs)[1]; 10 μM RN9893 (n = 6 donors), a specific inhibitor of transient receptor potential vanilloid type 4 (TRPV4) channels[3]. Effects were compared to water or DMSO vehicle controls. Strain-induced reduction in nuclear area (water vehicle control, p = 0.02; DMSO vehicle control, p = 0.05) was prevented by inhibition of stretch-activated ion channels ($GdCl_3$ inhibitor), specifically TRPV4 (RN9893 inhibitor), but not piezo inhibitor GsMTx4 (p = 0.0003). Nuclear areas of hMSCs recovered to pre-strained values 24 hours after CTS, with no significant residual effect from ion channel inhibition. p-values determined from linear modeling (ANOVA). Bars show mean ± s.e.m. (**b**) Distributions of nuclear areas between individual donors, used to make figure part (a). Box-whisker plot centre lines show means, bounds of box show quartiles, whiskers show data spread and outliers determined by the Tukey method. Minimum numbers of cells analysed per condition: donor d211 used ≥31 cells per condition; d224, ≥21; d226, ≥28; d230 ≥20; d144 ≥28; d007 ≥14.

Supplementary Fig. 3.

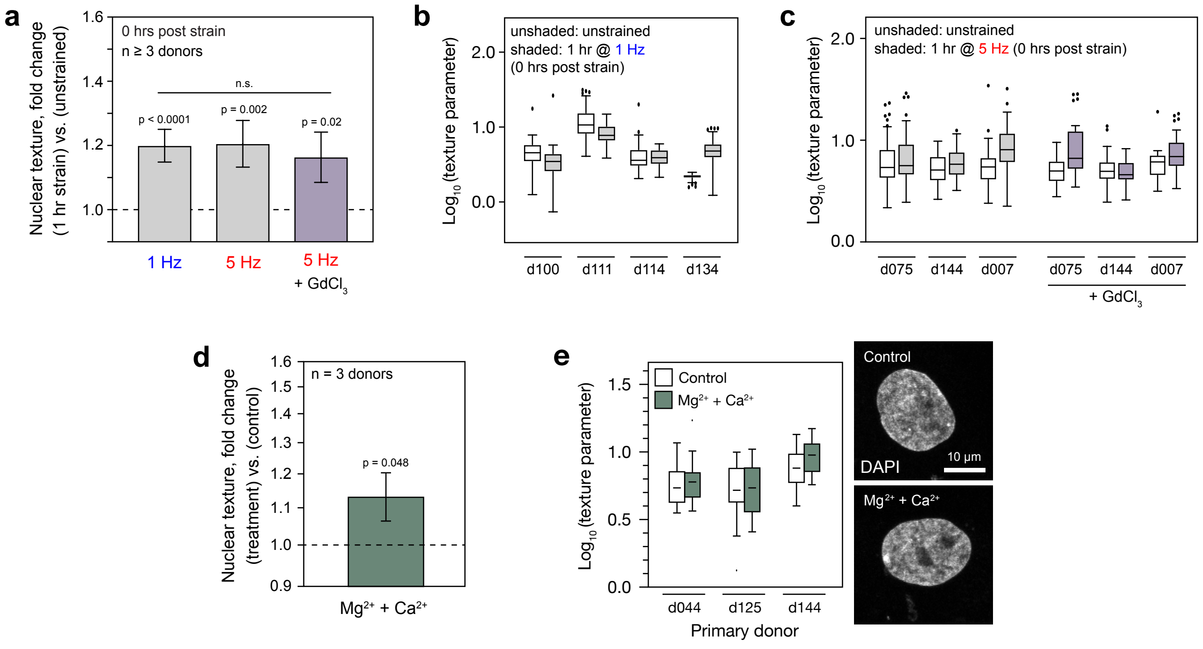

Supplementary Fig. 3. CTS-induced changes to texture of nuclear DAPI staining. (**a**) Changes to the texture of nuclear DAPI stains are indicative of chromatin condensation state[2, 4]. Plot shows that a parameter quantifying nuclear texture was increased by strain (1 hour, 0 – 4% strain at 1 Hz, $p < 0.0001$, $n = 4$ donors; or 2.6 – 6.2% strain at 5 Hz, $p = 0.002$, $n = 3$ donors) and that this increase was unaffected by 10 µM $GdCl_3$ treatment ($p = 0.02$, $n = 3$ donors), suggesting that CTS-induced nuclear texture changes occurred independently of stretch-activated ion channels. *p*-values determined from linear modeling (ANOVA). Bars show mean ± s.e.m. (**b**) Distributions of texture parameters, 1 Hz CTS (0 – 4% strain) vs. control, in 4 donor samples, used to make figure part (a). (**c**) Distributions of texture parameters, 5 Hz CTS (2.6 – 6.2% strain) vs. control, with 10 µM $GdCl_3$ treatment or vehicle, in 3 donor samples, used to make figure part (a). Minimum numbers of cells analysed per condition for figure parts (a)-(c): donor d100 used ≥33 cells per condition; d111, ≥38; d114, ≥41; d134, ≥87; d075, ≥24; d144, ≥28; d007, ≥21. Box-whisker plot centre lines show medians, bounds of box show quartiles, whiskers show data spread and outliers determined by the Tukey method. (**d**) The texture parameter was significantly increased in DAPI stained nuclei of hMSCs treated with calcium and magnesium ions (2 mM) ($p = 0.048$, $n = 3$ donors), conditions shown previously to result in chromatin condensation[5]. *p*-value determined from linear modeling (ANOVA). Bar shows mean ± s.e.m. (**e**) Distributions of texture parameters from 3 donor samples, used to make figure part (d); representative images of nuclei with chromatin condensation induced by treatment with bivalent ions. Minimum numbers of cells analysed per condition for figure parts (d), (e): donor d044 used ≥21 cells per condition; d125, ≥19; d144, ≥21. Box-whisker plot centre lines show means, bounds of box show quartiles, whiskers show data spread and outliers determined by the Tukey method.

Supplementary Fig. 4.

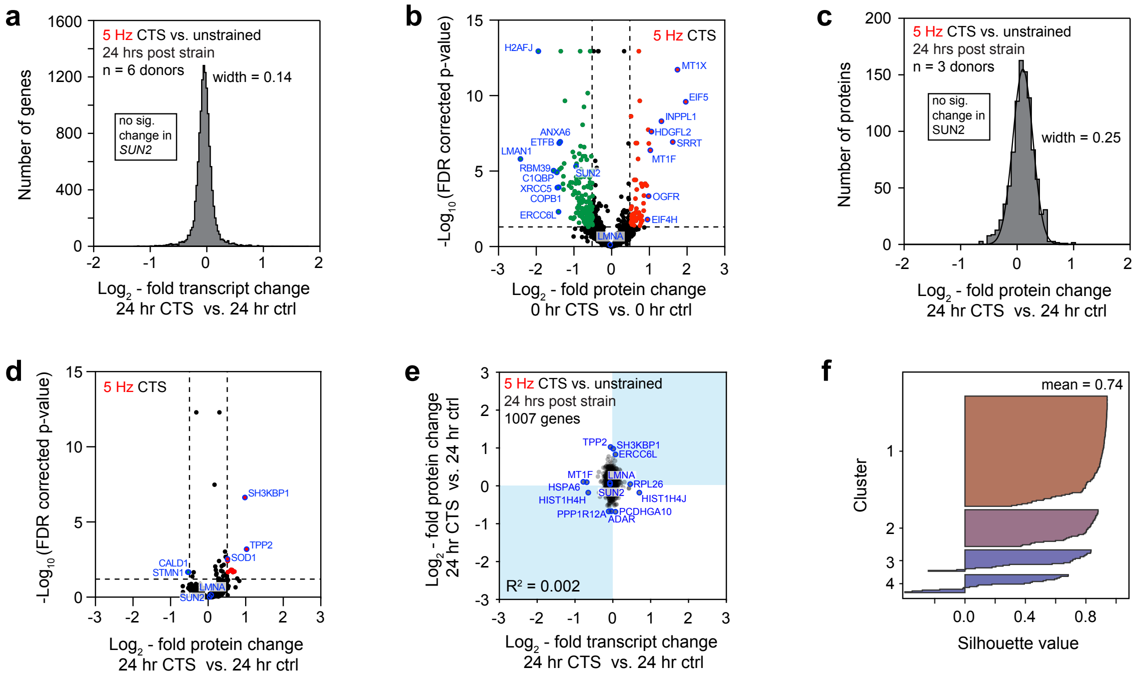

Supplementary Figure 4. Recovery of initial transcriptomic and proteomic states 24 hours after 5 Hz CTS. (**a**) Histogram showing that transcript levels were recovered 24 hours after high-intensity CTS (1 hour at 5 Hz, 2.6 – 6.2 % strain; n = 6 donors; Gaussian width = 0.14, compared to 0.21 immediately following CTS). See Supplementary Data 1 and 2 for transcriptomics data. (**b**) Volcano plot of changes to the proteome of hMSCs immediately following high-intensity CTS (1 hour at 5 Hz, 2.6 – 6.2 % strain; n = 3 donors). (**c**) Histogram and (**d**) volcano plot of data showing that protein levels were also recovered 24 hours after high-intensity CTS (Gaussian width of the histogram = 0.25, compared to 0.61 immediately following CTS), n = 3 donors. (**e**) Correlation plot between proteome and transcriptome 24 hours after high-intensity CTS (1007 genes quantified by RNA-seq and in proteomics by three-or-more peptides per protein; selected outlying genes/proteins of interest are annotated; R-squared = 0.002). All p-values were calculated using empirical Bayes-modified t-tests with Benjamini-Hochberg correction. See Supplementary Data 3 and 4 for proteomics data. (**f**) Data from proteomic analysis following CTS was split into four groups using a k-means clustering algorithm. The decision to use four clusters was based on the use of silhouette plots, wherein silhouette values denoted the similarity between the cluster centroid and each constitutive point within it. The number of clusters was chosen to maximize the average silhouette value.

Supplementary Fig. 5.

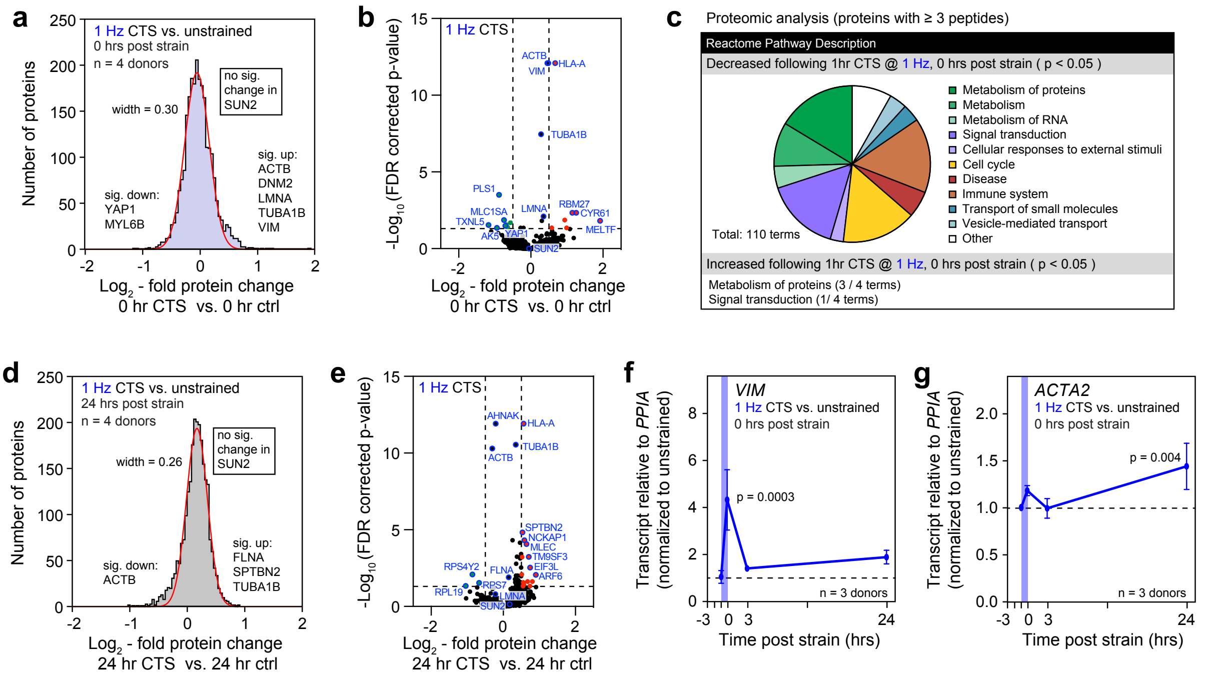

Supplementary Figure 5. Proteomic and transcriptomic changes to hMSCs subjected to 1 Hz CTS for one hour. (**a**) Histogram of proteomic changes quantified by mass spectrometry (MS) immediately following low intensity CTS (1 hour at 1 Hz, 0 – 4 % strain; *n* = 4 donors); data displayed as $\log_2$-fold change following CTS, versus unstrained controls, and shows proteins quantified by three-or-more peptides. A Gaussian fit to the distribution had a width of 0.30, suggesting a smaller perturbation to the proteome than that caused by 5 Hz CTS (Fig. 2c). There was no significant change in SUN2 protein levels. Cytoskeletal proteins ACTB, DNM2, TUBA1B and VIM, and the nuclear intermediate filament protein LMNA were upregulated with strain, while YAP1 and MYL6B were down regulated ($p < 0.05$). These changes were suggestive of cytoskeletal remodeling. (**b**) Volcano plot (significance versus fold change) of changes to the proteome of hMSCs immediately following low intensity CTS (1 hour at 1 Hz, 0 – 4 % strain; *n* = 4 donors). (**c**) Analysis of Reactome pathways significantly affected at the protein level following low intensity CTS ($p < 0.05$, including proteins quantified by three-or-more peptides). Pathways associated with cellular metabolism and signal transduction were significantly affected by 1 Hz CTS. (**d**) Histogram and (**e**) volcano plot of changes to the proteome 24 hours after low intensity CTS (1 hour at 1 Hz, 0 – 4 % strain; *n* = 4 donors); a Gaussian fit to the histogram had a width of 0.26, suggesting a return to a baseline state when compared to the distribution immediately following CTS. However, there was evidence of persistent remodeling of the cytoskeleton, with levels of FLNA, SPTBN2 and TUBA1B upregulated, while ACTB was downregulated ($p < 0.05$). SUN2 remained unresponsive. All *p*-values were calculated using empirical Bayes-modified t-tests with Benjamini-Hochberg correction. Peptide lists, protein fold-changes and *p*-values are provided in Supplementary Data 8 and 9. (**f**) Transcript levels of vimentin (*VIM*) in hMSCs, *n* = 3 donors, subjected to low intensity CTS (0 – 4% strain at 1 Hz for 1 hour). A significant spike was observed immediately following strain, suggestive of cytoskeletal remodeling ($p = 0.0003$). (**g**) Transcript levels of alpha-actin-2 (aortic smooth muscle actin, *ACTA2*) following low intensity CTS; levels were significantly increased after 24 hours ($p = 0.004$), *n* = 3 donors. In figure parts (f) and (g), measurements were made relative to housekeeping gene *PPIA* (peptidyl-prolyl cis-trans isomerase A) and normalized to unstrained controls; significance determined by ANOVA testing.

Supplementary Fig. 6.

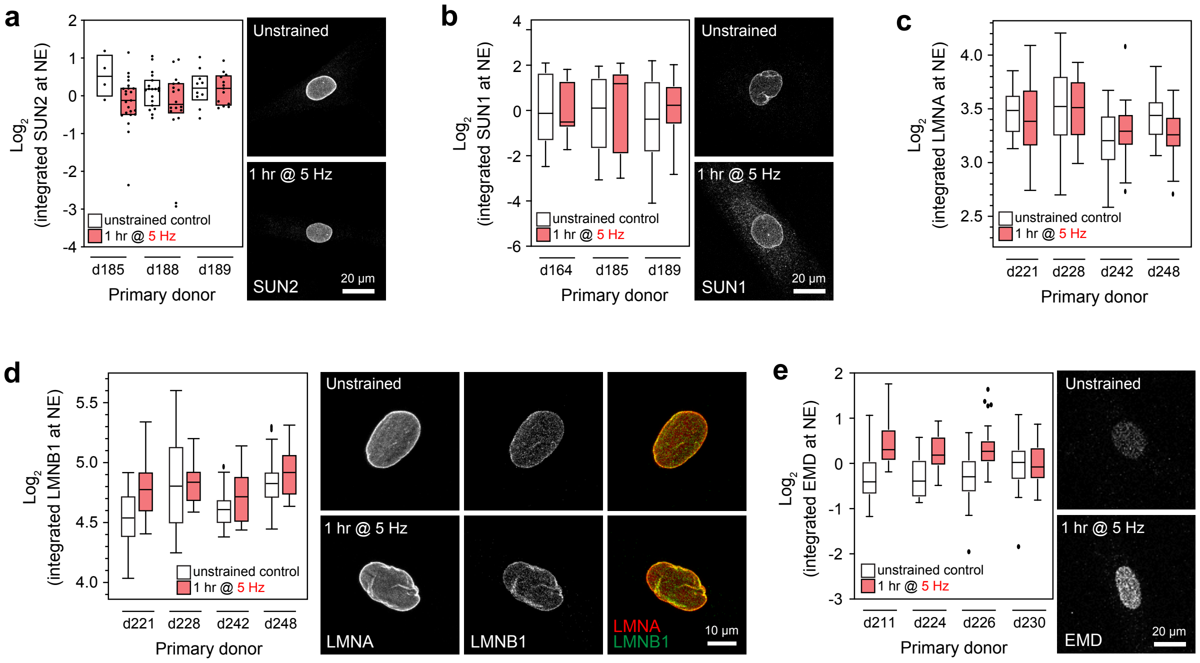

Supplementary Figure 6. Quantitation of proteins at the nuclear envelope (NE) in hMSCs following 5 Hz CTS. (**a**) Immunofluorescence (IF) quantification of SUN domain-containing protein 2 (SUN2) at the NE in hMSCs subject to high-intensity CTS (1 hour at 5 Hz, 2.6 – 6.2 % strain) and compared to unstrained controls; n = 3 donors; donor d185, ≥4 cells analysed per condition; donor d188, ≥18 cells per condition; donor d189, ≥10 cells per condition. All data points are shown; bars show quartile ranges with centre line indicating the median. (**b**) IF quantification of SUN domain-containing protein 1 (SUN1) at the NE in hMSCs subject to high-intensity CTS and compared to unstrained controls; n = 3 donors; donor d164, ≥24 cells analysed per condition; donor d185, ≥21 cells per condition; donor d189, ≥17 cells per condition. (**c**) IF quantification of lamin-A/C (LMNA) at the NE in hMSCs subjected to high-intensity CTS, compared to unstrained controls. (**d**) IF quantification of lamin-B1 (LMNB1) at the NE in hMSCs subject to high-intensity CTS and compared to unstrained controls. Analysis for (c) and (d) had n = 4 donors; donor d221, ≥22 cells analysed per condition; donor d228, ≥16 cells per condition; donor d242, ≥21 cells per condition; donor d248, ≥19 cells per condition. (**e**) Emerin (EMD) quantified by IF at the NE in hMSCs subjected to high-intensity CTS and compared to unstrained controls; n = 4 donors; donor d211, ≥31 cells analysed per condition; donor d224, ≥22 cells per condition; donor d226, ≥28 cells per condition; donor d230, ≥32 cells per condition. In figure parts (a)-(e), the location of the NE was determined from DAPI staining. Images show representative nuclei. Linear modeling (ANOVA) to account for donor variation showed CTS to significantly decrease SUN2 (p = 0.03), while LMNB1 and EMD were increased (p = 0.001 and p < 0.0001, respectively), see Fig. 4b. Images show representative nuclei. In box-whisker plots in figure parts (b)-(e) centre lines show medians, bounds of box show quartiles, whiskers show data spread and outliers determined by the Tukey method.

Supplementary Figure 7.

a

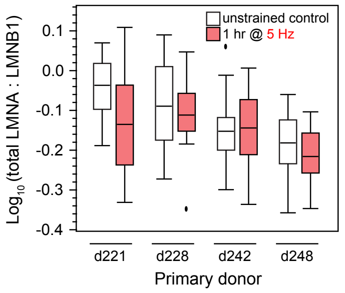

b

	Stiff vs. soft	1 Hz CTS vs. static	5 Hz CTS vs. static
Cytoplasmic spread area	Increased	Increased	No change
Nuclear spread area	Increased	No change	Decreased
LMNA abundance	Increased*	Increased	No change
LMNA : LMNB ratio	Reported to scale with tissue stiffness*	Not determined	Decreased
LMNA phosphorylation	Decreased pS22, pS390, pS404, pT424*	No modifications detected	Increased pS22, pS390, pS392, pS636
LMNA mBBr labeling	No change in Cys-522 labeling*	Not determined	Increase in Cys-522 labeling
SUN2 abundance	Reported to scale with tissue stiffness*	No change	Decreased

* (Swift et al. Science, 2013)

Supplementary Figure 7. Effect of CTS on ratio of total lamin-A/C to lamin-B1. (**a**) Ratio of total lamin-A/C (LMNA) to lamin-B1 (LMNB1) quantified by immunofluorescence in hMSCs immediately following CTS (n = 4 donors, cell images and numbers as in Supplementary Figure 6c, d). CTS treatment decreased total LMNA : B1 to 0.92 ± 0.03 (mean ± s.e.m., p = 0.007; determined from linear modeling (ANOVA)). Centre lines show medians, bounds of box show quartiles, whiskers show data spread and outliers determined by the Tukey method. (**b**) Comparison of the effects of static and dynamic mechanical stimulation on the morphology and nuclear lamina of hMSCs. LMNA was increased in abundance on stiff vs. soft substrates, attributed to reduced phosphorylation allowing assembly of the lamina to be reinforced[6]. This reinforcement was also considered to avert unfolding of the mechano-sensitive LMNA Ig-domain, preventing mBBr labeling of the LMNA Cys-522 site. In contrast, we have found that 5 Hz CTS desensitised the lamina to many aspects of mechanically-induced lamina remodeling, an observation that we consider to be consistent with SUN2-mediated nuclear decoupling: LMNA abundance was not affected by 5 Hz CTS, while phosphorylation and mBBr labelling were increased. Although characterised to a lesser extent, the response of hMSCs to 1 Hz CTS was perhaps more akin to the response to increased stiffness.

Supplementary Figure 8.

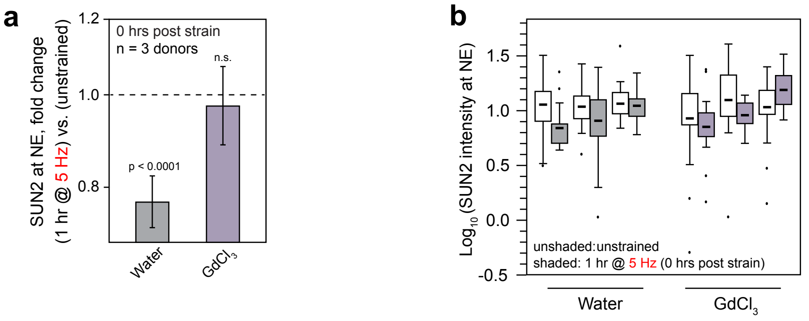

Supplementary Figure 8. Ion channel inhibition prevents CTS-induced loss of SUN2 from the nuclear envelope (NE). (**a**) Changes to SUN2 levels measured by immunofluorescence at the NE in hMSCs immediately following CTS (1 hour, 2.6 – 6.2% strain at 5 Hz; n = 3 donors) with and without ion channel inhibition (10 μM $GdCl_3$), normalized to unstrained controls. Functional ion channels were required for significant down-regulation of SUN2 at the NE following CTS ($p < 0.0001$, two-tailed student t test); bars show mean ± s.e.m. determined from linear modeling. (**b**) Donor-to-donor variation in the response of SUN2 levels at the NE of hMSCs following CTS (1 hour, 2.6 – 6.2% strain at 5 Hz) with ion channel inhibition (10 μM $GdCl_3$) (data used to make figure part (a)). n = 3 donors: donor d007, ≥21 cells analysed per condition; donor d075, ≥24 cells per condition; donor d144, ≥28 cells per condition. Centre lines show means, bounds of box show quartiles, whiskers show data spread and outliers determined by the Tukey method.

Supplementary Fig. 9.

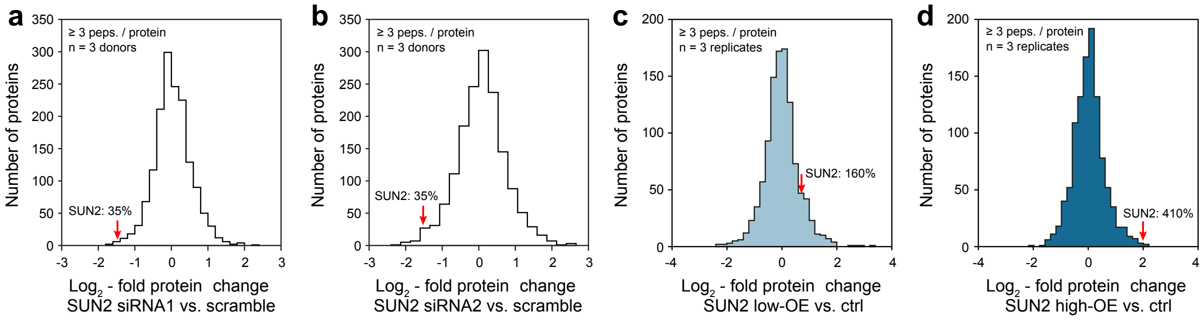

Supplementary Figure 9. Cellular proteomes of hMSCs following knockdown (KD), overexpression (OE) and rescue of SUN2 expression levels. Histograms of changes to intracellular protein levels, determined by mass spectrometry (MS) in primary hMSCs treated with (**a**) siRNA1 targeting SUN2; (**b**) siRNA2 targeting SUN2, relative to scrambled controls (*n* = 3 donors; plots show proteins quantified by at least three peptides-per-protein). Both siRNAs were found to reduce SUN2 levels to 35% of scrambled controls. Histograms of changes to intracellular protein levels, determined by MS in an immortalised hMSC line[7] virally infected with a plasmid to enable SUN2 OE to (**c**) low (160% of control levels) and (**d**) high levels (410% of control levels), relative to vehicle-only controls (*n* = 3 replicates, plots show proteins quantified by at least three peptides-per-protein). See Supplementary Data 10-13 for peptide lists, protein fold-changes and *p*-values associated with figure parts (a)-(d). All *p*-values were calculated using empirical Bayes-modified t-tests with Benjamini-Hochberg correction.

Supplementary Figure 10.

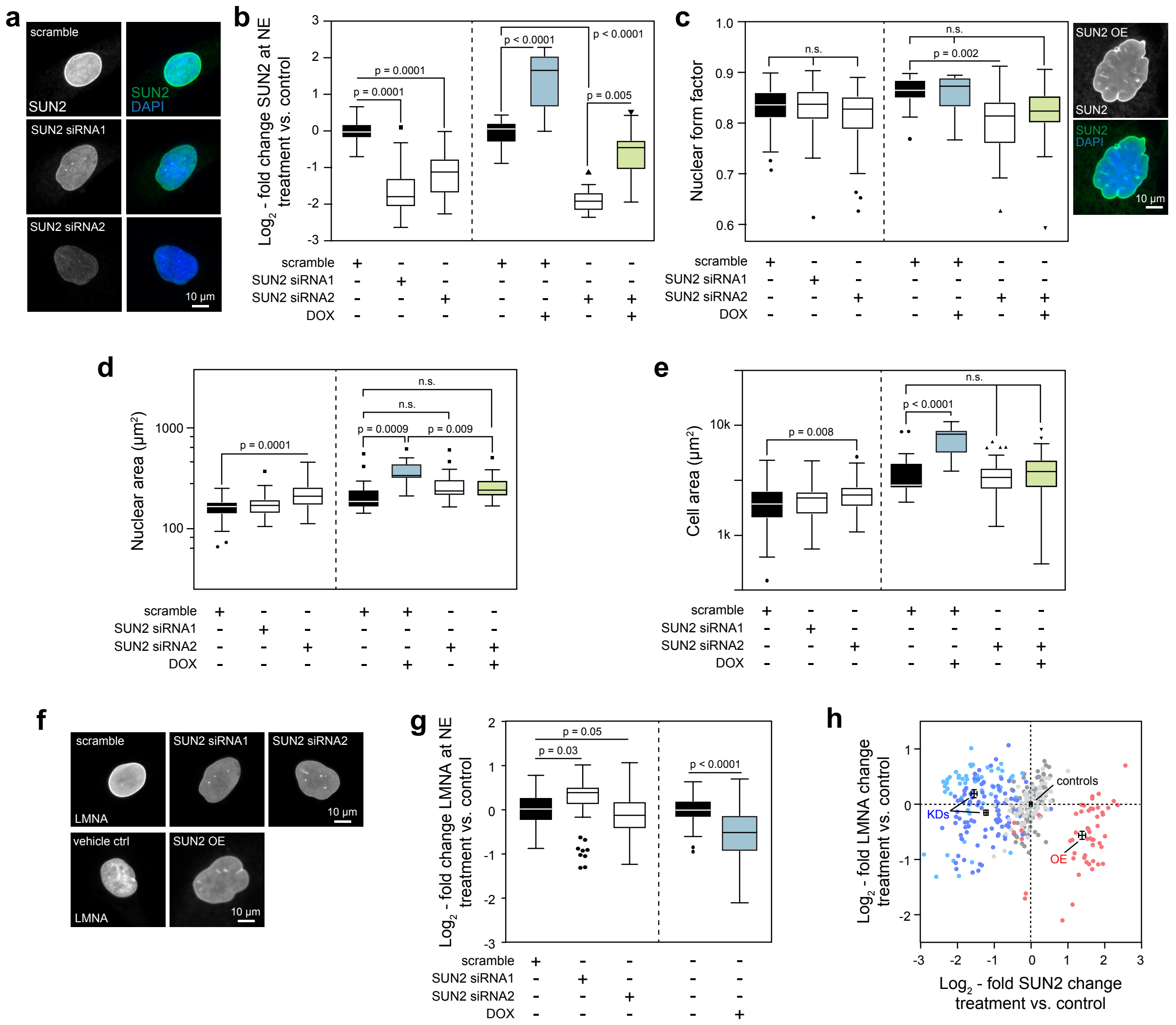

Supplementary Figure 10. Morphology of hMSCs following knockdown (KD), overexpression (OE) and rescue of SUN2 expression levels. (**a**) Example immunofluorescence (IF) images of immortalised hMSCs subjected to SUN2 KD (siRNA1, siRNA2 or scrambled control), with staining for SUN2 and DAPI. Quantification of (**b**) SUN2 at the NE by IF, (**c**) nuclear form factor (area multiplied by 4π, divided by square of perimeter), (**d**) nuclear area and (**e**) cell area in immortalised hMSCs subjected to SUN2 KD (siRNA1 or siRNA2 compared to a scrambled control) or SUN2 OE/rescue (doxycycline (DOX)-induced OE, siRNA2 or DOX-induced OE + siRNA2 compared to a scrambled control). SUN2 KD reduced SUN2 levels at the NE ($p \leq 0.0001$), OE increased SUN2 ($p < 0$ .0001) and rescue returned SUN2 levels towards those of the control (SUN2 levels increased vs. KD, $p = 0.005$). In one replicate, SUN2 KD with siRNA2 caused a decrease in form factor vs. scrambled control ($p = 0.002$). Deformation of the nuclear membrane was observed in some OE cells (see example image with figure part (c) showing SUN2 and DAPI staining), and has been reported previously[8]. SUN2 KD with siRNA2 increased nuclear area vs. control in one replicate ($p = 0.0001$); SUN2 OE increased nuclear area ($p = 0.0009$), but this was reversed by rescue ($p = 0.009$). Cell area was also increased in a replicate SUN2 KD with siRNA2 ($p = 0.008$) and by SUN2 OE ($p < 0.0001$); this was restored in the rescue. (**f**) Representative images of nuclei stained against lamin-A/C (LMNA) in immortalised hMSCs following SUN2 KD or OE. (**g**) Analysis of LMNA protein levels quantified by IF at the NE in immortalised hMSCs with SUN2 KD (increased LMNA levels in siRNA1 SUN2 KD vs. scrambled control, $p = 0.03$; decreased LMNA levels in siRNA2 SUN2 KD vs. scrambled control, $p = 0.05$), and SUN2 OE (LMNA levels decreased vs. control, $p < 0.0001$). (**h**) Scatter plot of LMNA levels quantified by IF at the NE with SUN2 KD, and DOX-induced SUN2 OE in an immortalised MSC line (data points indicate the nuclei of individual cells). All box-whisker plot centre lines show medians, bounds of box show quartiles, whiskers show data spread and outliers determined by the Tukey method. *P*-values were determined from one-way ANOVA tests followed by Dunnett's multiple comparison tests. Figure parts (b)-(e) contain analysis of ≥11 hMSCs analysed per condition; (g) and (h), ≥54 hMSCs per condition.

Supplementary Fig. 11.

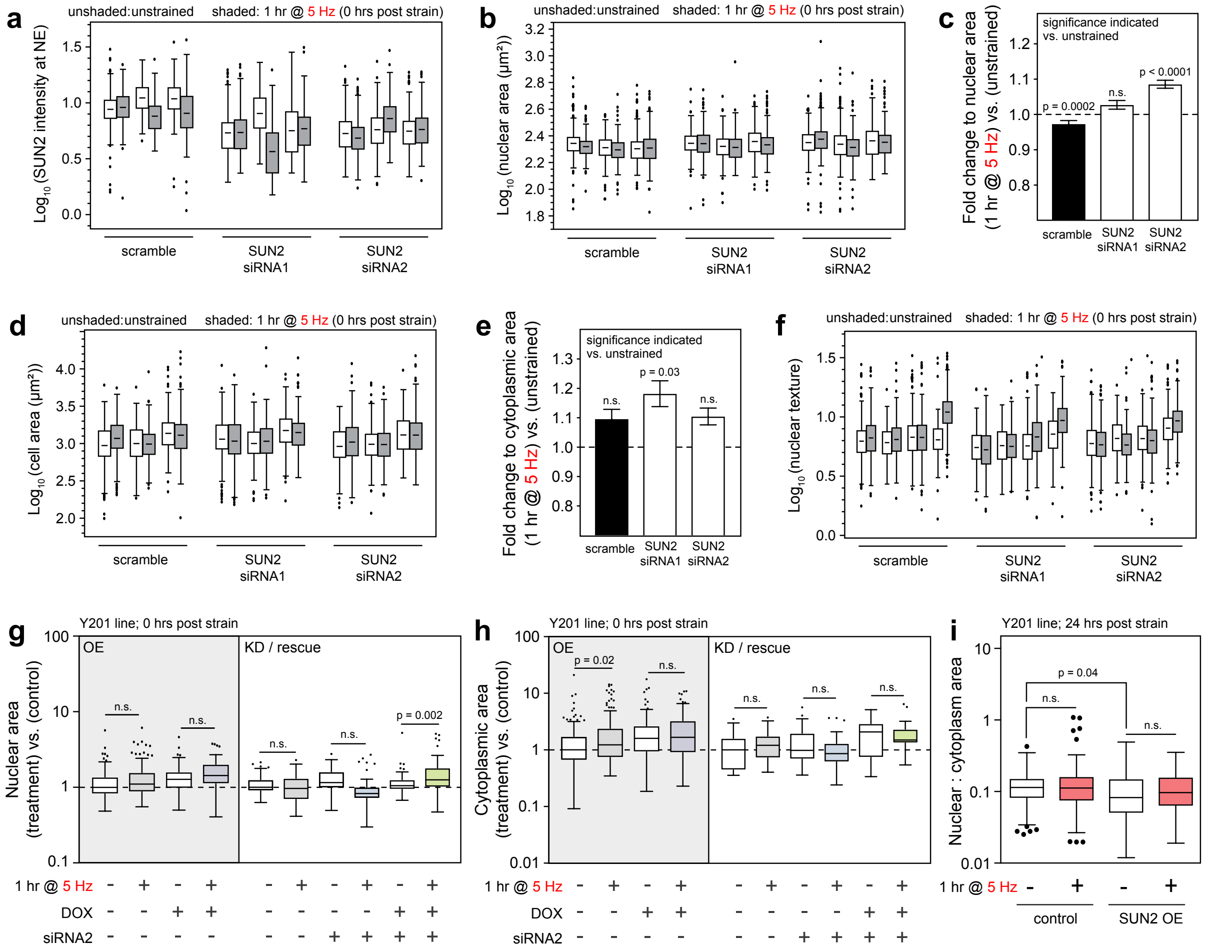

Supplementary Figure 11. Response to 5 Hz CTS in hMSCs following KD or OE of SUN2. (**a**) Donor-to-donor variation in SUN2 at the nuclear envelope, quantified by immunofluorescence, in primary hMSCs immediately following CTS at 5 Hz (1 hour, 2.6 – 6.2% strain), comparing SUN2 KD to scrambled controls. (**b**) Donor-to-donor variation in nuclear areas of primary hMSCs immediately following CTS at 5 Hz, comparing SUN2 KD to scrambled controls. (**c**) Fold changes to nuclear areas, summarised from figure part (b). Nuclear area was decreased following CTS ($p = 0.0002$). siRNA1 KD of SUN2 prevented a change in nuclear area following CTS. siRNA2 SUN2 KD led to an increase in nuclear area following CTS ($p < 0.0001$). (**d**) Donor-to-donor variation in cell spread areas of primary hMSCs immediately following CTS at 5 Hz, comparing SUN2 KD to scrambled controls. (**e**) Fold changes to cell areas, summarised from figure part (d). Cell area increased following siRNA2, but not siRNA1, KD of SUN2 plus CTS ($p = 0.03$). Figure parts (c) and (e) show mean ± s.e.m., $n = 3$ primary donors. (**f**) Donor-to-donor variation in nuclear texture of primary hMSCs immediately following CTS at 5 Hz, comparing SUN2 KD to scrambled controls. In figure parts (a), (b), (d) and (f), shaded bars indicate cells analysed immediately following CTS, unshaded bars show controls; box-whisker plot centre lines show means, bounds of box show quartiles, whiskers show data spread and outliers determined by the Tukey method; ≥174 cells analysed per donor, per condition. (**g**) Nuclear areas of immortalised hMSCs with SUN2 OE, SUN2 KD (siRNA2) and induced rescue of SUN2, immediately following CTS at 5 Hz, normalised to (-/-/-) controls. SUN2 rescue resulted in an increase in nuclear area following CTS ($p = 0.002$). No effect was observed in any other treatments. (**h**) Cytoplasmic areas of immortalised hMSCs with SUN2 OE, SUN2 KD (siRNA2) and induced rescue of SUN2, immediately following CTS at 5 Hz, normalised to (-/-/-) controls. Cell area increased following CTS ($p = 0.02$), but cell area remained unchanged for all other treatments. Figure parts (g), (h) and (i) had ≥35 cells per condition. (**i**) Ratios of nuclear to cytoplasmic areas in immortalised hMSCs with SUN2 OE 24 hours post CTS. Differences in nuclear to cytoplasmic area ratios induced by CTS in OE cells (Fig. 8e) were lost 24 hours after straining. A decrease in the ratio of nuclear to cytoplasmic area remained for unstrained cells with SUN2 OE ($p = 0.04$). In figure parts (g)-(i), box-whisker plot centre lines show medians, bounds of box show quartiles, whiskers show data spread and outliers determined by the Tukey method. All p-values determined from Dunnett's multiple comparison tests.

Supplementary Figure 12.

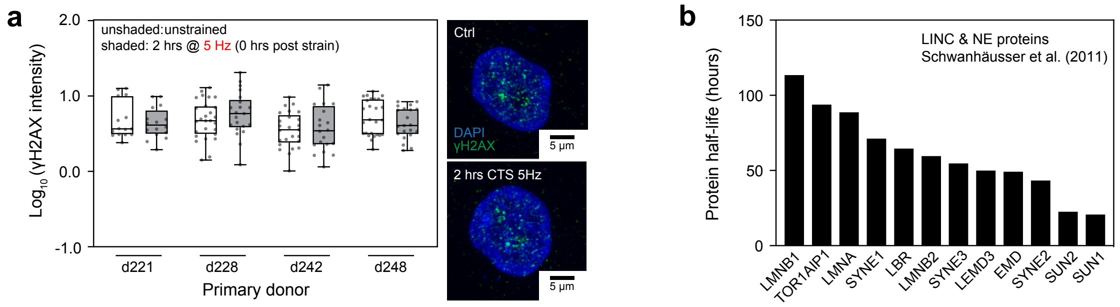

Supplementary Figure 12. Phospho-γ H2AX staining and half-lives of nuclear envelope proteins. (**a**) Donor-by-donor analysis of γ H2AX phosphorylation assessed in primary hMSCs immediately following CTS (2 hour, 2.6 – 6.2%, 5 Hz). n = 4 primary donors; minimum 15 cells analysed per condition for donor d221; ≥22 cells, d228; ≥20 cells, d242; ≥21 cells, d248. Images show representative nuclei with γH2AX and DAPI staining. Box-whisker plot centre lines show medians, bounds of box show quartiles, whiskers show data spread and outliers determined by the Tukey method. (**b**) Plot of mean half-lives of linker of nucleoskeleton and cytoskeleton (LINC) complex and nuclear envelope (NE) proteins, determined in mouse fibroblasts by Schwanhausser *et al.*[9]. The turnover of SUN proteins was found to be faster than other NE and LINC components.

Supplementary Table 1

Summary of numbers of cells analysed in each experiment.

Main Figures	Supplementary Figures	Cell donor ID	Range of cell numbers analysed per condition
1a, d		d269	12 - 32
		d270	42 - 52
		d296	32 - 48
		d305	62 - 81
		d310	64 - 109
		d320	68 - 86
1b, c, e-g	1c, f	d100	14 - 46
		d111	13 - 57
		d114	27 - 91
		d134	27 - 93
	1f	d175	33 - 69
4b	6b	d164	24 -33
	6a, b	d185	4 - 25
	6a	d188	18 - 19
	6a, b	d189	10 - 21
	6e	d211	31 - 34
	6c, d; 7a	d221	22 - 23
	6e	d224	22 - 23
	6e	d226	28 - 30
	6c, d; 7a	d228	16 - 23
	6e	d230	32 - 39
	6c, d; 7a	d242	21 - 24
	6c, d; 7a	d248	19 - 23
	8a, b	d007	21 - 32
	8a, b	d075	24 - 60
	8a, b	d144	28 - 41
4c, d		d211	53 - 55
		d297	24 - 27
		d299	20 -21
7b, c, d	10b-e	Y201 (immortalised)	11 - 33
	10g, h	Y201 (immortalised)	54 - 93
8a, b, c	11a-f	d211	280 - 912
		d224	174 - 319
		d226	179 - 285
		d230	182 - 350
8d, e	11g, h	Y201 (immortalised)	21 - 159
8f		Y201 (immortalised)	17 - 80
	11i	Y201 (immortalised)	76 - 183
9a	12a	d221	15 - 16
		d228	22 - 27
		d242	20 - 25
		d248	21 - 26
9b		Y201 (immortalised)	29 - 37
9c		Y201 (immortalised)	27 - 80

Supplementary References

1. Martinac, B. The ion channels to cytoskeleton connection as potential mechanism of mechanosensitivity. *Biochim. Biophys. Acta-Biomembranes* **1838**, 682-691 (2014).
2. Heo, S.J. *et al.* Biophysical regulation of chromatin architecture instills a mechanical memory in mesenchymal stem cells. *Sci. Rep.* **5**, 14 (2015).
3. Wei, Z.L. *et al.* Identification of orally-bioavailable antagonists of the TRPV4 ion-channel. *Bioorg. Med. Chem. Lett.* **25**, 4011-4015 (2015).
4. Irianto, J. *et al.* Osmotic challenge drives rapid and reversible chromatin condensation in chondrocytes. *Biophys. J.* **104**, 759-769 (2013).
5. Chalut, K.J. *et al.* Chromatin decondensation and nuclear softening accompany nanog downregulation in embryonic stem cells. *Biophys. J.* **103**, 2060-2070 (2012).
6. Swift, J. *et al.* Nuclear lamin-A scales with tissue stiffness and enhances matrix-directed differentiation. *Science* **341**, 1240104 (2013).
7. James, S. *et al.* Multiparameter analysis of human bone marrow stromal cells identifies distinct immunomodulatory and differentiation-competent subtypes. *Stem Cell Rep.* **4**, 1004-1015 (2015).
8. Donahue, D.A. *et al.* SUN2 overexpression deforms nuclear shape and inhibits HIV. *J. Virol.* **90**, 4199-4214 (2016).
9. Schwanhausser, B. *et al.* Global quantification of mammalian gene expression control. *Nature* **473**, 337-342 (2011).
