# Supplementary material for: Nuclear decoupling is part of a rapid protein-level cellular response to high-intensity mechanical loading
Source: Nat Commun. 2019 Sep 12;10:4149. doi: 10.1038/s41467-019-11923-1 (PMC6742657; doi:10.1038/s41467-019-11923-1)
Supplement: Supplementary file 3 — Description of Additional Supplementary Files [file 41467_2019_11923_MOESM3_ESM.pdf]

Description of Additional Supplementary Files

File Name: Supplementary Data 1
Description: RNA-Seq CTS at 5 Hz -- Raw data

File Name: Supplementary Data 2
Description: RNA-Seq CTS at 5 Hz -- Fold changes to protein-coding genes

File Name: Supplementary Data 3
Description: Proteomics CTS at 5 Hz -- Peptide list from Progenesis QI

File Name: Supplementary Data 4
Description: Proteomics CTS at 5 Hz -- Fold-changes to proteins

File Name: Supplementary Data 5
Description: Proteomics CTS at 5 Hz -- Fold-changes to mBBr labelling

File Name: Supplementary Data 6
Description: Proteomics CTS at 5 Hz -- Fold-changes to phosphorylation sites

File Name: Supplementary Data 7
Description: Proteomics CTS at 5 Hz -- Fold-changes to oxidation sites

File Name: Supplementary Data 8
Description: Proteomics CTS at 1 Hz -- Peptide list from Progenesis QI

File Name: Supplementary Data 9
Description: Proteomics CTS at 1 Hz -- Fold-changes to proteins

File Name: Supplementary Data 10
Description: Proteomics SUN2 KD -- Peptide list from Progenesis QI

File Name: Supplementary Data 11
Description: Proteomics SUN2 KD -- Fold-changes to proteins

File Name: Supplementary Data 12
Description: Proteomics SUN2 OE -- Peptide list from Progenesis QI

File Name: Supplementary Data 13
Description: Proteomics SUN2 OE -- Fold-changes to proteins
