# Supplementary material for: Nuclear decoupling is part of a rapid protein-level cellular response to high-intensity mechanical loading
Source: Nat Commun. 2019 Sep 12;10:4149. doi: 10.1038/s41467-019-11923-1 (PMC6742657; doi:10.1038/s41467-019-11923-1)
Supplement: Supplementary file 17 — Reporting Summary [file 41467_2019_11923_MOESM17_ESM.pdf]

nature research

Corresponding author(s): JOE SWIFT

Last updated by author(s): Jul 3, 2019

Reporting Summary

Nature Research wishes to improve the reproducibility of the work that we publish. This form provides structure for consistency and transparency in reporting. For further information on Nature Research policies, see Authors & Referees and the Editorial Policy Checklist .

Statistics

For all statistical analyses, confirm that the following items are present in the figure legend, table legend, main text, or Methods section.

n/a	Confirmed	
☐	☒	The exact sample size (*n*) for each experimental group/condition, given as a discrete number and unit of measurement
☐	☒	A statement on whether measurements were taken from distinct samples or whether the same sample was measured repeatedly
☐	☒	The statistical test(s) used AND whether they are one- or two-sided<br>*Only common tests should be described solely by name; describe more complex techniques in the Methods section.*
☐	☒	A description of all covariates tested
☐	☒	A description of any assumptions or corrections, such as tests of normality and adjustment for multiple comparisons
☐	☒	A full description of the statistical parameters including central tendency (e.g. means) or other basic estimates (e.g. regression coefficient) AND variation (e.g. standard deviation) or associated estimates of uncertainty (e.g. confidence intervals)
☒	☐	For null hypothesis testing, the test statistic (e.g. *F*, *t*, *r*) with confidence intervals, effect sizes, degrees of freedom and *P* value noted<br>*Give P values as exact values whenever suitable.*
☐	☒	For Bayesian analysis, information on the choice of priors and Markov chain Monte Carlo settings
☒	☐	For hierarchical and complex designs, identification of the appropriate level for tests and full reporting of outcomes
☐	☒	Estimates of effect sizes (e.g. Cohen's *d*, Pearson's *r*), indicating how they were calculated

Our web collection on statistics for biologists contains articles on many of the points above.

Software and code

Policy information about availability of computer code

Data collection	Xcalibur (version 4.1.31.9, Thermo Scientific); LAS X (version 3.5, Leica).
Data analysis	Progenesis QI (version 4.1, Nonlinear Dynamics); Excel for Mac (version 16, Microsoft); Mathematica (version 11, Wolfram Research); GraphPad (version 8, Prism); Igor Pro (version 6.37, Wavemetrics); MatLab (version R2015a, MathWorks); ImageJ (version 2.0.0; National Institutes of Health); CellProfiler (version 2.1.1, Broad Institute).<br>The BayesENproteomics code used to process MS data is available to download from GitHub:<br>https://www.github.com/VenkMallikarjun/BayesENproteomics

For manuscripts utilizing custom algorithms or software that are central to the research but not yet described in published literature, software must be made available to editors/reviewers. We strongly encourage code deposition in a community repository (e.g. GitHub). See the Nature Research guidelines for submitting code & software for further information.

Data

Policy information about availability of data

All manuscripts must include a data availability statement. This statement should provide the following information, where applicable:

- Accession codes, unique identifiers, or web links for publicly available datasets
- A list of figures that have associated raw data
- A description of any restrictions on data availability

Proteomics data have been deposited to the ProteomeXchange Consortium via the PRIDE partner repository with the identifiers: PXD012863, PXD012873, PXD012948, PXD012949 and PXD013287. RNA-Seq data is available via EMBL-EBI ArrayExpress with identifier E-MTAB-7925.

Field-specific reporting

Please select the one below that is the best fit for your research. If you are not sure, read the appropriate sections before making your selection.

- [x] Life sciences
- [ ] Behavioural & social sciences
- [ ] Ecological, evolutionary & environmental sciences

For a reference copy of the document with all sections, see nature.com/documents/nr-reporting-summary-flat.pdf

Life sciences study design

All studies must disclose on these points even when the disclosure is negative.

Sample size	Experimental sample sizes were chosen in order to achieve statistical power while acknowledging the limitations to the availability of primary human tissue. A minimum of 3 human donors were used per study, with up to 6 donors included when sample availability permitted. Rigorous statistical testing across all experiments was used to derive significance.
Data exclusions	No data were excluded from the analysis.
Replication	All experiments were replicated in line with the number of donors used.
Randomization	Cells from human donors were chosen based on their availability at the time of experimentation. Sample order was randomized before analysis by mass spectrometry.
Blinding	The investigators were not blinded. Analysis of microscopy images was performed by a computer algorithm (CellProfiler).

Reporting for specific materials, systems and methods

We require information from authors about some types of materials, experimental systems and methods used in many studies. Here, indicate whether each material, system or method listed is relevant to your study. If you are not sure if a list item applies to your research, read the appropriate section before selecting a response.

Materials & experimental systems

n/a	Involved in the study	
	x	Antibodies
	x	Eukaryotic cell lines
x		Palaeontology
x		Animals and other organisms
	x	Human research participants
x		Clinical data

Methods

n/a	Involved in the study	
x		ChIP-seq
x		Flow cytometry
x		MRI-based neuroimaging

Antibodies

Antibodies used	Anti- SUN1, Sigma, HPA008461, polyclonal<br>Anti- SUN2, Sigma, HPA001209, polyclonal<br>Anti- LMNA/C, Santa Cruz Biotechnology, sc-7292, monoclonal – clone 636<br>Anti- LMNB1, Abcam, ab16048, polyclonal<br>Anti- Emerin, Leica Microsystems, EMERIN-CE, monoclonal – clone 4G5)<br>Anti- phospho-histone H2AX (S139) (γH2AX) Merck, 05-636, monoclonal – clone JBW301<br>AlexaFluor-488 goat anti-mouse, ThermoFisher Scientific, A11029, polyclonal<br>AlexaFluor-594 donkey anti-rabbit, ThermoFisher Scientific, A21207, polyclonal
Validation	Data acquired from antibody methods were supported by orthogonal mass spectrometry proteomics experiments.

Eukaryotic cell lines

Policy information about cell lines

Cell line source(s)	The immortalised mesenchymal stem cell line Y201 was a gift from Professor Paul Genever (University of York, UK).
Authentication	We have confirmed that the cells can be induced to undergo adipogenesis and osteogenesis. A full characterisation of the cell line can be found in James et al. (Stem Cell Rep. 2015).

Mycoplasma contamination	All cell lines used in the laboratory are routinely tested for mycoplasma contamination.
Commonly misidentified lines (See ICLAC register)	None.

Human research participants

Policy information about studies involving human research participants

Population characteristics	Human mesenchymal stem cells (hMSCs) were isolated from the bone marrow (knee and hip) of male and female donors.
Recruitment	Participants were recruited who were undergoing surgical procedures to knee and hip. This is likely to bias towards older participants. Samples were anonymised, with only donor age, sex and cell source site made available. Sample sizes were not large enough to establish statistical links to these variables.
Ethics oversight	Informed written consent was obtained from donors. Experiments followed guidelines and regulations in accordance with the WMA Declaration of Helsinki and the UK Human Tissue Authority. All work was performed with approval from the NHS Health Research Authority National Research Ethics Service (approval number 10/H1013/27) and the University of Manchester.

Note that full information on the approval of the study protocol must also be provided in the manuscript.
